# Relationship between Mediterranean Dietary Polyphenol Intake and Obesity

**DOI:** 10.3390/nu10101523

**Published:** 2018-10-17

**Authors:** Sara Castro-Barquero, Rosa M. Lamuela-Raventós, Mónica Doménech, Ramon Estruch

**Affiliations:** 1 Institut d’Investigacions Biomèdiques August Pi i Sunyer (IDIBAPS), 08036 Barcelona, Spain; sacastro@clinic.cat (S.C.-B.); mdomen@clinic.cat (M.D.); 2 Department of Medicine, Faculty of Medicine and Health Sciences, University of Barcelona, 08036 Barcelona, Spain; 3 Department of Nutrition, Food Science and Gastronomy, XaRTA, INSA-UB, School of Pharmacy and Food Science, University of Barcelona, 08028 Barcelona, Spain; lamuela@ub.edu; 4 CIBEROBN Fisiopatología de la Obesidad y Nutrición, Instituto de Salud Carlos III, 28029 Madrid, Spain; 5 Department of Internal Medicine Institut d’Investigacions Biomèdiques August Pi Sunyer (IDIBAPS), Hospital Clinic, University of Barcelona, 08036 Barcelona, Spain

**Keywords:** dietary intake, catechins, resveratrol, olive oil, wine, BMI

## Abstract

Obesity is a multifactorial and complex disease defined by excess of adipose mass and constitutes a serious health problem. Adipose tissue acts as an endocrine organ secreting a wide range of inflammatory adipocytokines, which leads to systemic inflammation, insulin resistance, and metabolic disorders. The traditional Mediterranean diet is characterized by a high phenolic-rich foods intake, including extra-virgin olive oil, nuts, red wine, vegetables, fruits, legumes, and whole-grain cereals. Evidence for polyphenols’ effect on obesity and weight control in humans is inconsistent and the health effects of polyphenols depend on the amount consumed and their bioavailability. The mechanisms involved in weight loss in which polyphenols may have a role are: activating β-oxidation; a prebiotic effect for gut microbiota; inducing satiety; stimulating energy expenditure by inducing thermogenesis in brown adipose tissue; modulating adipose tissue inhibiting adipocyte differentiation; promoting adipocyte apoptosis and increasing lipolysis. Even though the intake of some specific polyphenols has been associated with body weight changes, there is still no evidence for the effects of total polyphenols or some polyphenol subclasses in humans on adiposity.

## 1. Introduction

The global overweightness and obesity epidemic is increasing at an alarming rate and constitutes a serious global public health problem, affecting over 27.5% of the worldwide adult population and 47.1% of children [1]. Between 1980 and 2013, the worldwide prevalence of overweight and obese individuals increased from 857 million to 2.1 billion [1]. There is some evidence that the obesity epidemic is leveling off in some populations, although the prevalence of excess weight remains high in many countries of the world. The health consequences associated with obesity have been widely recognized: overall mortality, cardiovascular disease (CVD), hypertension, type 2 diabetes mellitus (T2DM), hyperlipidemia, stroke, cancer, osteoarthritis, chronic kidney disease, and gynecological problems, among others [2]. The medium-to-long-term consequences of obesity lead to rendering the health system unsustainable and, consequently, an urgent priority must be given to finding solutions for this issue that should be based on the best scientific evidence available.

Obesity is a multifactorial complex disease defined by excess of adipose mass, which occurs through adipocyte hypertrophy and hyperplasia [3]. The adipose tissue is an endocrine organ that secretes a wide variety of inflammatory adipocytokines, such as tumor necrosis factor alpha (TNF-α), interleukin-6 (IL-6), resistin, leptin, and adiponectin. Visceral adiposity is associated with a higher production of these inflammatory adipocytokines, leading to systemic inflammation, insulin resistance, and several obesity-related metabolic disorders [4]. This inflammation due to obesity can be reversed with weight loss, which causes a reduction in fat mass and proinflammatory adipokines. Moreover, the intake of foods rich in bioactive compounds such as omega-3 fatty acids and polyphenols have been described to decrease low-degree inflammation [3].

## 2. The Mediterranean Diet

The link between adherence to the traditional Mediterranean diet (MedDiet) and the risk of cardiovascular disease (CVD) are mediated by several mechanisms, including reduction in low-degree inflammation [5,6,7], high plasma concentration of adiponectin, improvement of endothelial function [8], diminution of oxidative stress [9], low concentration of atherogenic lipoproteins, and lower levels of oxidized low-density lipoprotein (LDL) particles [10]. The high-density lipoprotein (HDL) functionality was also improved by the MedDiet. Cholesterol efflux capacity, specifically the HDL esterification index and HDL antioxidant and anti-inflammatory capacity, and vasoprotective effects inducing nitric oxide synthesis by endothelial cells are increased [11]. Furthermore, there are other inflammatory biomarkers related to CVD and atherosclerotic process that may be modulated by lifestyle, such as C-reactive protein (CRP), IL-6, and homocysteine [7,12].

**Figure 1:**
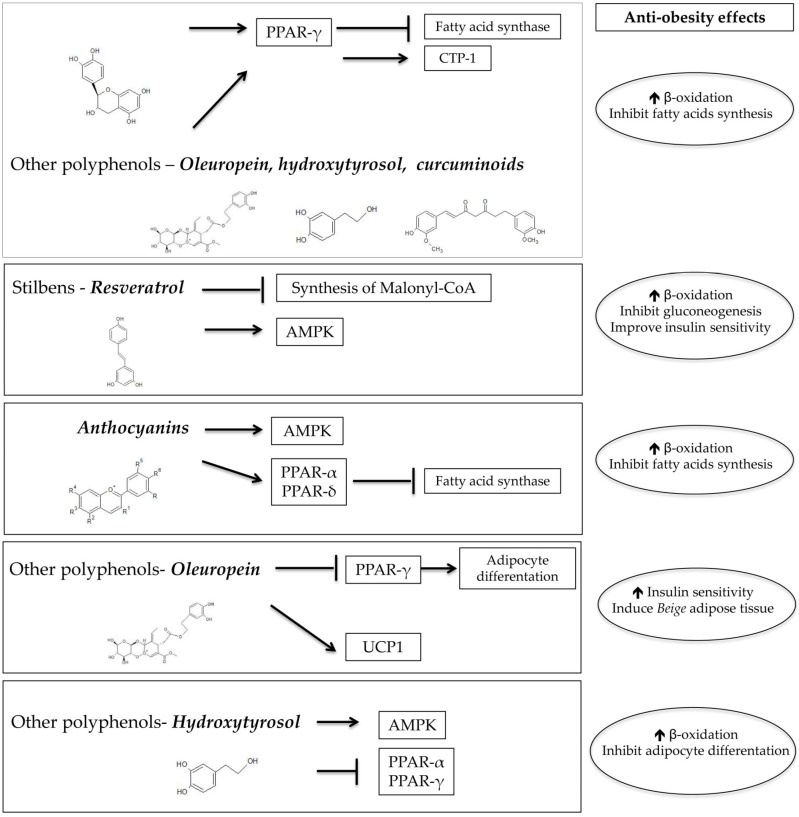
Molecular mechanisms of polyphenols involved in obesity. PPAR-γ: peroxisome proliferator-activated receptor gamma; CTP-1: tricarboxylate transport protein 1; AMPK: 5′-adenosine monophosphate-activated protein kinase; PPAR-α: peroxisome proliferator-activated receptor alpha; PPAR-δ: peroxisome proliferator-activated receptor delta; PPAR-γ: peroxisome proliferator-activated receptor gamma; → activation; → inhibition; and ↑ increase. ADC/ChemSketch (Advanced Chemistry Development, Inc., Toronto, ON, Canada) software was employed for chemical structures.

The MedDiet is characterized by a high intake of phenolic compounds, which are present in the main key foods of this dietary pattern: extra-virgin olive oil (EVOO), nuts, red wine, legumes, vegetables, fruits, and whole-grain cereals. Phenolic compounds, usually called polyphenols (Figure 1) [13], are important candidates responsible for the beneficial effects of the MedDiet. A continuous and prolonged polyphenol intake is related to blood pressure and adiposity lowering effects, improvements in lipid profile, and also anti-inflammatory effects, which all act as CVD protectors [14].

### Mediterranean Diet and Weight Loss

Although the long-term health benefits of the MedDiet are well established, its efficacy for weight loss at ≥12 months in overweight or obese individuals remains controversial. A systematic review of five randomized clinical trials (RCTs) [15] studied the effect of the MedDiet on weight loss in overweight or obese individuals comparing MedDiet interventions with low-fat diets, a low-carbohydrate diet, and the American Diabetes Association (ADA) diet. In this review, the MedDiet showed greater weight loss than the low-fat diets (range of the mean values: −4.1 to −10.1 kg vs. −2.9 to −5.0 kg), but similar weight loss compared with the other two interventions (range of the mean values: −4.1 to −10.1 kg vs. −4.7 to −7.7 kg). Epidemiological evidence for the association between the adherence to a traditional MedDiet with reduction of body weight and waist circumference is unclear. In 2011, Esposito et al. published a meta-analysis of 16 RCTs, which shows that a greater adherence to the MedDiet causes more weight loss as compared with a control diet [5]. Moreover, in none of the 16 RCTs was MedDiet adherence correlated with weight gain. Many components of the MedDiet may favor weight loss due to the abundance of plant-based foods, which provide high dietary fiber intake with a low energy density and low glycemic load. However, the effect of the MedDiet on body weight was greater in association with an energy-restricted MedDiet plan (−3.88 kg) or physical activity improvements (−4.01 kg) [16].

Huo et al. studied the effect of a Mediterranean-style diet on T2DM patients in terms of glycemic control, weight loss, and cardiovascular risks factors. Body mass index (BMI) was decreased in participants who followed the MedDiet (mean difference, −0.29 kg/m^2^; 95% CI, −0.46 to −0.12) compared with those in the control diets [16].

## 3. Dietary Polyphenol Intake

The effects of polyphenols depend on the amount and absorption of dietary polyphenols. Thus, to highlight the health benefits of polyphenols in humans, it is necessary to know the polyphenol content of the foods and the polyphenol subclasses’ composition. Typically, polyphenol intake is currently evaluated using data extracted from food frequency questionnaires (FFQs). Recently, polyphenol intake has been measured using analysis of different biomarkers, mainly phase II enzyme-conjugated polyphenol metabolites, which are metabolites present in the bloodstream and urine and fecal samples. Unfortunately, there are thousands of potential biomarkers of polyphenol intake and there is no consensus yet [17]. On the other hand, Tresserra-Rimbau et al. studied the effect of dietary polyphenol intake on CVD, calculating the polyphenol consumption by matching FFQ data with the Phenol-Explorer database [14]. In this context, the effect of gut microbiota has to be considered, as it metabolizes part of the dietary polyphenols and its metabolism can modify their absorption, bioavailability, and biological activity. The interindividual variability in gut microbiota, which determines polyphenol absorption, can explain the variety of health effects in the mentioned studies.

### Polyphenol Intake in the Mediterranean Countries

The intake of dietary polyphenols and the main food sources depends on the dietary pattern and the native foods of each region, as described in Table 1. In the case of Mediterranean countries, the European Prospective Investigation into Cancer (EPIC) Nutrition cohort described the differences among the polyphenol intake of the European regions, estimating individual polyphenols and subclasses [18]. The estimation of polyphenol intake was performed by 24-h dietary recall of 36,027 adults, and the phenolic compounds data was obtained using the Phenol-Explorer database. Interestingly, the Mediterranean countries (including Spain, Greece, Italy, and the south of France) showed the lowest intake of total polyphenols (around 1011 mg/day) compared with non-Mediterranean countries and the United Kingdom (around 1284 and 1521 mg/day, respectively) [18]. Nevertheless, the profile of polyphenol subclasses was very different: Mediterranean countries showed the highest intake of stilbenes and flavonoids (49–62% of total polyphenols), followed by phenolic acids (34–44%). In relation to the main food sources, polyphenols in Mediterranean countries come mainly from coffee, fruits (the main source of flavonoids, representing 45% of the intake), wine, and vegetables oils (representing 26% of lignans intake), whereas in the non-Mediterranean countries, polyphenols come from coffee, tea, and wine (40.9%, 17.4%, and 4.6% of total polyphenols, respectively) [18].

Another cohort from France, called SUpplémentation en VItamines et Minéraux AntioXydants (SU.VI.MAX), quantified the polyphenol intake by 24-h dietary records and the Phenol-Explorer database in 4942 subjects. The mean total polyphenol intake (TPI) was 1193 mg/day, with hydroxycinnamic acids being the highest consumed polyphenol subclass, followed by proanthocyanidins [19]. The main food sources of hydroxycinnamic acids were coffee, potatoes, and apples, whereas for proanthocyanidins, were fruits, cocoa products, and red wine.

An observational study focusing on the nutritional habits characterizing the Mediterranean lifestyle, performed in Sicily in southern Italy, named the Mediterranean healthy Eating, Aging, and Lifestyle study (MEAL), estimated the polyphenol intake of 2044 subjects by FFQs and the Phenol-Explorer database. The main objective of the study was to describe the polyphenol intake differentiating the subjects by their level of adherence to the MedDiet, as measured by the MEDI-LITE score [20]. Additionally, Godos et al. described the intake of polyphenol subclasses and the major food sources in the MEAL study population [21]. Total polyphenol intake was 664 mg/day, of which the main intakes by subclass were phenolic acids, followed by flavonoids (363 and 259 mg/day, respectively). Nuts were the main food source of polyphenols, accounting for around 28% of total polyphenol intake, followed by coffee, cherries, red wine, and tea. Despite the fact that the adherence to the Mediterranean diet was high, the intake of total polyphenols was lower than the other areas described. The study concluded that the most consumed subclasses were flavonoids among the individuals with the highest adherence to the MedDiet, with fruits, vegetables, and red wine being the main food contributors [22].

The PREDIMED cohort (PREvención con DIeta MEDiterránea), comprised of a Spanish population at high cardiovascular risk, studied the effect of dietary polyphenol intake and the incidence of cardiovascular events [14]. Tresserra-Rimbau et al. described the intake of polyphenol subclasses’ intake and the major food sources of the PREDIMED study subjects also using FFQs and the Phenol-Explorer database. Similar to the Italian population, the main intakes by subclass were flavonoids (443 mg/day), followed by phenolic acids (304 mg/day) [23]. Fruits were the main total polyphenols contributor, accounting for around 44%. Within the flavonoids, flavanols were strongly related to CVD prevention (HR = 0.4 (0.23–0.72)) and were mostly consumed from red wine (32%) and apples (31%) [14]. This study concluded that a higher intake of flavanols was associated with a 60% reduction of cardiovascular event and mortality risk. Despite the fact that the main phenolic acids subclass consumed was hydroxycinnamic acids, the intake of hydroxybenzoic acids was related to a lower incidence of CVD (HR = 0.47 (0.26–0.86)). It should be pointed out that increased intake of lignans was also related to CVD prevention (HR = 0.51 (0.30–0.86)), even though their intake was lower than 1 mg/day.

The main key foods of the MedDiet in the PREDIMED cohort were EVOO and nuts. EVOO and olives provide around 11% of the total polyphenol intake. The phenolic profile of EVOO and olives is unique, with 98% of the polyphenols being inside the ‘other phenolic acids’ and ‘other polyphenols’ subclasses. Among these subclasses, oleuropein is associated with antidiabetes, antiatherosclerosis, and anti-inflammation properties [24]. This characteristic phenolic profile has resulted in health benefits, a claim which was recognized by the European Food Safety Authority (EFSA) [25].

## 4. Antiobesity Effects of Dietary Polyphenols

Evidence for polyphenols’ effect on obesity and weight control in humans is inconsistent due to the heterogeneity among study design, study populations, intervention period, and polyphenol supplements. These potential effects are summarized in Table 2. Some intervention clinical trials with polyphenol-enriched foods, such as an apple juice, showed a significant reduction in body fat mass but not in body weight, BMI, or waist circumference [26]. However, a recent double-blinded, randomized, parallel clinical trial conducted in 17 type 1 obesity participants (BMI between 30.1 and 33.3 kg/m^2^) with a polyphenol supplement of 370 mg of total polyphenols showed a significant reduction in body weight, BMI, and waist and hip circumference compared with a placebo group after 12 weeks of intervention [27]. Moreover, only a few studies have studied the relationship between TPI from diet and weight control. Guo et al. [28] analyzed the association between body weight and TPI using a urine biomarker in a high cardiovascular risk population in a long-term study. After five years of follow-up, they showed an inverse association between total polyphenol excretion (TEP) and BMI, body weight, and waist circumference [28].

Similarly, a study conducted in the Mediterranean area demonstrated that higher dietary intake of flavonoids is inversely associated with an excess of weight and obesity [29]. Studies conducted in non-Mediterranean areas have shown an effect of polyphenol intake on weight control, but other clinical trials did not find any relationship between polyphenol intake and weight loss or changes in body composition (CITA).

A longitudinal study from a Netherlands cohort that included 4280 participants aged 55–69 years over 14 years of follow-up showed an association between a higher flavonoids intake and a lower increase in body mass index (BMI) in women (*p* < 0.05) [30]. Within the flavonoids, catechins are related with benefits in anthropometric parameters and body composition. More evidence that includes some studies with green tea extracts rich in catechins, epigallocatechin gallate (EGCG), showed a significant reduction in body weight, waist circumference, body fat mass, and visceral and subcutaneous fat [31]. Based on a meta-analysis of 11 studies, Hursel et al. concluded that catechin or an EGCG–caffeine mixture contained in green tea had a minimal effect on weight loss and weight loss maintenance [31]. Therefore, the clinical significance of the small changes seen in the body composition parameters indicates that green tea has no significant effect on weight loss and weight loss maintenance [32].

Resveratrol, a phenolic compound found in grapes, red wine, and some berries, also has potential antiobesity effects by inhibiting adipocyte differentiation and decreasing proliferation, mediated by adipocyte apoptosis and decreasing lipogenesis, promoting lipolysis and β-oxidation [30]. However, evidence about the effect of resveratrol intake on weight loss and weight loss maintenance is limited and the effects only seem to be achieved through dietary supplementation. Tome-Carneiro et al. performed several randomized, parallel, dose–response, placebo-controlled studies with a grape supplement rich in resveratrol and other grape polyphenols [33,34]. The effects were statistically significant for CVD risk factors: reduction in LDL-cholesterol, oxidized LDL, and thrombogenic plasminogen activator inhibitor type 1 (PAI-1), and increase in adiponectin and anti-inflammatory cytokines; however, they were not significant for adiposity parameters. Thus, the antiobesity potential and the optimal dose of resveratrol remain to be studied.

Despite the fact that the spice turmeric is not a characteristic food of the MedDiet, curcumin, a yellow-colored polyphenol from the curcuminoids subclass, is known for its health benefits such as anti-inflammatory, anticarcinogenesis, antiobesity, antiangiogenesis, and antioxidant activities [35]. The antiobesity properties of curcumin are similar to resveratrol, through inhibiting adipocyte differentiation, lipogenesis, reducing proinflammatory cytokines’ synthesis in the adipose tissue, and promoting β-oxidation [35]. Similar to resveratrol, clinical trials to investigate the antiobesity properties of curcumin are limited. Ramirez-Bosca reported improvements in serum lipid profile through an increase in HDL-cholesterol and Apo A, as well as a decrease in LDL-cholesterol, ApoB, and the ApoB/ApoA ratio [36] with a supplement dose of 10 mg of a curcumin extract daily over 30 days.

Evidence from in vitro and experimental models suggests the potential effects of polyphenols on obesity, obesity-related inflammation, and other metabolic disorders. These studies show significant reduction of body weight by increasing basal metabolic rate, increasing β-oxidation, lowering triglycerides synthesis, and improving insulin sensitivity. Obese individuals have been reported to be more dependent on glucose oxidation rather that fat oxidation [37]. The mechanisms involved in weight loss where polyphenols may have a role are: inducing satiety; stimulating energy expenditure by inducing thermogenesis in brown adipose tissue; modulating adipose tissue by inhibiting adipocyte differentiation and promoting adipocyte apoptosis; modulating lipolysis; and activating β-oxidation [38]. Relative to metabolic disorders, an in vitro study about the effect of white tea EGCG showed improvements in cellular glucose metabolism mediated by glucose transporters (GLUTs) and a potential hypocholesterolemic effect stimulating LDL receptor binding activity [39].

### Gut Microbiota and Prebiotic Potential of Dietary Polyphenols

The gut microbiota is, nowadays, strongly associated with several complex diseases, especially when this microbiota is imbalanced, also known as dysbiosis. This dysbiosis may be disrupted by lifestyle, such as excessive sanitation, diet, sedentarism, antibiotics, and so forth. Related to the topic of this review, the microbiota has a role in the host’s metabolism, energy extraction, fat deposition, inflammatory status, gut barrier integrity, and also satiety [40]. The roles of the molecules generated from bacterial fermentation are crucial to establishing the causal relevance of the gut microbiota and health benefits.

Short-chain fatty acids (SCFAs) are formed from the fermentation of oligosaccharides, proteins, and peptides [41], with the main SCFA products being acetate, propionate, and butyrate. The consumption of complex carbohydrates from fruits and vegetables is associated with higher microbial production of SCFAs [42]. The contribution of SCFA products against obesity has been linked to decreasing weight gain by preventing fat accumulation [43,44,45]. Fernandes et al. showed that obese subjects present higher SCFA products in stool samples than lean subjects because of the differences in their colonic fermentation [42]. The before-mentioned SCFA main products display different mechanisms to induce satiety: butyrate acts on intestinal cells, increasing GLP-1 production [46], and propionate increases intestinal gluconeogenesis [45], both pathways leading to improvements in glucose homeostasis and increasing satiety.

Besides the microbial products, the gut microbiota is crucial for the metabolism and degradation of some other compounds. Branched-chain amino acids (BCAAs) are elevated in obesity and T2DM, which are contributing to the development of obesity-related insulin resistance. A reduction in BCAA level is strongly correlated with improvements in insulin sensitivity, more so than weight loss [47]. Interestingly, the composition of the gut bacteria, specifically the invasion of *Bacteroides* spp., may improve the efficiency of BCAA degradation [48].

Nevertheless, the main tool to balance the gut microbiota is diet. This notion is promoting the use of prebiotics, which are mainly dietary components such as nondigestible carbohydrates. Other dietary compounds not absorbed by the small intestine, such as polyphenols, are accumulated in the large intestine, thus being exposed to the enzymatic activities of the gut microbiota [49]. In vitro studies suggested that polyphenols may act as prebiotics by enhancing the growth of beneficial bacteria such as *Lactobacillus* spp. and *Bifidobacterium* spp. [50]. Related to the SCFAs, polyphenols from plum were reported to decrease fecal SCFAs in obese rats and, consequently, prevent weight gain in association with the changes in the bacterial composition of the gut microbiota by increasing *Faecalibacterium* spp., *Lactobacillus* spp., and *Bacteroidetes* spp. proliferation [50]. The potential prebiotic effect of proanthocyanidin on *Akkermansia muciniphila* is well described by Anhê et al. [51]. The pathways through which proanthocyanidins can enhance *Akkermansia* proliferation are: increasing mucus secretion to the intestinal lumen by goblet cells; proanthocyanidins and other polyphenols may use free oxygen radicals in the intestinal lumen, creating an environment only favorable for strict aerobic species; antimicrobial effects of polyphenols may help to degrade competitive bacteria of *Akkermansia*.

Relative to proanthocyanidins, a dietary supplement of grape seed extract in six female pigs caused a change in the distribution of the microbiota, increasing *Lachnospiraceae*, unclassified *Clostridales*, *Lactobacillus*, and *Ruminococcacceae* [52]. The same experimental models used by Quifer-Rada et al. described the molecular mechanisms of the potential hypocholesterolemic effects of proanthocyanidins shown in human studies [53]. The grape seed extract increases biliary excretion and reduces micellar solubility, which translates to a higher excretion of cholesterol in feces [54].

## 5. Mechanism Involved

Catechins, mainly green tea EGCC, promote β-oxidation by regulating the expression in adipose tissue of peroxisome proliferator-activated receptor gamma (PPAR-γ) and fatty acid synthase (FAS), while increasing the levels of CPT-1, a protein that facilitates the transport of fatty acids to the mitochondria, which is a limiting step for β-oxidation [54].

In the case of resveratrol, its involvement in regulating β-oxidation has been studied by increasing 5′-adenosine monophosphate-activated protein kinase (AMPK) activity through preventing the degradation of intracellular cyclic adenosine monophosphate (cAMP) [55]. The AMPK function is to regulate glucose transport and fatty acid metabolism. Therefore, its activation may lead to fatty acid oxidation and suppression of hepatic gluconeogenesis as well as improvements in insulin sensitivity. Other studies revealed that resveratrol could mediate the expression of PPAR-γ [56] or promote β-oxidation by inhibiting the synthesis of malonyl-CoA [57], which is a precursor and promoter of fatty acid synthesis.

Curcumin contains polyphenols, and there is substantial evidence about its effectiveness in stimulating β-oxidation, inhibiting fatty acid synthesis, and decreasing fat storage [38]. The molecular pathways are similar to EGCG in the upregulation of CPT-1, but also entail the reduction of lipid biosynthesis by the downregulation of fatty acid synthesis enzymes [58].

Within the flavonoids, anthocyanins have been reported as having a role as antiobesity agents. Anthocyanins are widely found in fruits, such as apples with peel, strawberries, blueberries, blackberries, and blood oranges. To induce fatty acid oxidation, the postulated pathways are the modulation of AMPK synthesis and regulation of the expression of genes participating in β-oxidation [59].

Regarding EVOO polyphenols, tyrosol derivates, such as oleuropein, are involved in energy metabolism and adiposity [60], reducing the expression of PPAR-γ, compromising adipocyte differentiation, and improving insulin sensitivity [61]. Another interesting mechanism studied by Oi-Kano et al. in experimental models showed an increase in uncoupling protein 1 (UCP1) expression, which translates to the formation of “beige” adipose tissue, leading to a decrease of visceral fat mass [62]. Hydroxytyrosol and its derivatives constitute around 90% of the total polyphenol content of EVOO [63]. In vitro studies reported that hydroxytyrosol downregulates the expression of PPAR-α and -γ, which is translated to a reduction in adipocyte size [64]. Additionally, an increase in AMPK and lipase (hormone-sensitive and phosphorylated lipase) was observed in adipocytes exposed to hydroxytyrosol [65]. Furthermore, these effects were not reported to have an impact on body weight and adiposity in humans [65].

There are several mechanisms of action involved and each polyphenol presents different pathways, as shown in Figure 1.

However, more randomized clinical trials are needed to verify if the ability of polyphenols to act as antioxidants and anti-inflammatory mediators, through suppressing the effects of oxidative stress and inflammation, can be translated to antiobesity effects.

## 6. Conclusions

The characteristic phenolic profile of the MedDiet differs from other dietary patterns, especially in the Mediterranean countries, where EVOO and olives are food sources that provide unique phenolic compounds with health benefits.

The health effects of polyphenols depend on the amount consumed and their bioavailability, which is low, and systemic concentrations of phenolic compounds may reach the millimolar range. As previously mentioned, the gut microbiota might be the most remarkable factor for the absorption and metabolism of dietary polyphenols. Moreover, bioavailability can be also modulated by the effects of culinary techniques, dietary patterns, or alteration of phase I/II metabolism by pharmacological or dietary agents.

However, the essential step towards the understanding of the protective effects of polyphenols against overweightness, unhealthy body composition, obesity-related inflammatory processes, and metabolic syndrome status is to estimate their consumption by dietary recalls (through 24-h dietary recall or FFQs) or other methods such as measurements of urine concentration of key polyphenols, in order to identify the compounds most likely to provide the greatest protection.

Even though the intake of some specific polyphenols has been associated with body weight improvements, there is still no evidence for the effects of total polyphenols or some polyphenol subclasses. Further randomized controlled trials are needed to confirm the promising protective effects of polyphenols on weight gain, obesity, and CVD. This research field might be useful for setting food and health counselling goals for overweightness and obesity, and additionally, to establish dietary recommendations for individuals and population groups and desired minimum levels of polyphenol intake.

## Figures and Tables

**Table 1:** Profile of the dietary polyphenol subclasses’ intake among the Mediterranean countries.

Mediterranean Area	Polyphenol Subclass (% of TPI) ^a^	Main Food Sources (% of TPI) ^a^
Spain, Greece, Italy, and south of France [17]	Phenolic acids (49), flavonoids (45), other polyphenols (0.6), stilbenes, and lignans (<0.7)	Coffee (36), fruits (25), red wine (10)
France [18]	Phenolic acids (54), flavonoids (42)	Coffee (44), tea (7), apples (7), red wine (6)
Spain [23]	Flavonoids (54), phenolic acids (37), other polyphenols (8.7), stilbenes, and lignans (<0.3)	Coffee (18), oranges (16), apples (12), olives and olive oil (11), red wine (6)
Sicily (Italy) [20,21]	Phenolic acids (53), flavonoids (37), lignans (0.4), stilbenes (0.3)	Nuts (28), coffee (7), red wine (6), tea (5)

TPI; Total polyphenol intake. ^a^ Dietary polyphenol intake was determined by the Phenol-Explorer Database (http://phenol-explorer.eu/, accessed on July 2018) for all the areas described.

**Table 2:** Potential health benefits on body weight by Mediterranean diet polyphenols.

Phenolic Compound	Potential Health Benefits	References
Total polyphenols	↓ Body weight, BMI, and waist and hip circumferences	[26]
Total polyphenols	Prebiotic effect ↑ *Lactobacillus* spp., *Bifidobacterium* spp., *Faecalibacterium* spp., and *Bacteroidetes* spp. proliferation	[49]
Total polyphenols	↓ SFCAs excretion	[49]
Flavonoids	↓ BMI	[27]
Epigallocatechin gallate (EGCG) and green tea extracts	↓ Body weight, fat mass, and visceral and subcutaneous fat	[31]
Proanthocyanidins	↑ Proliferation of the *Akkermansia muciniphila* spp.	[50]
Proanthocyanidins	↓ Total cholesterol levels ↑ Biliary excretion and micellar solubility	[52]
Resveratrol	↓ Adipocyte proliferation ↓ Lipogenesis ↑ Lipolysis and β-oxidation	[28]

^1^ BMI: Body mass index; SFCAs: Short-chain fatty acids; ↓ significant decrease; and ↑ significant increase.
